# Evidence of impaired macroautophagy in human degenerative cervical myelopathy

**DOI:** 10.1038/s41598-022-15158-x

**Published:** 2022-07-13

**Authors:** Sam S. Smith, Adam M. H. Young, Benjamin M. Davies, Hitoshi Takahashi, Kieren S. J. Allinson, Mark R. N. Kotter

**Affiliations:** 1 Academic Neurosurgery Unit, Department of Clinical Neurosurgery, University of Cambridge, Cambridge, UK; 2 WT MRC Cambridge Stem Cell Institute, Anne McLaren Laboratory, University of Cambridge, Cambridge, UK; 3 School of Clinical Medicine, University of Cambridge, Cambridge, UK; 4 Department of Pathology, Brain Research Institute, Niigata University, Niigata, Japan; 5 Cambridge University Hospitals NHS Foundation Trust and the Cambridge Brain Bank, Cambridge, UK

**Keywords:** Neuroscience, Autophagy, Spinal cord diseases

## Abstract

Degenerative cervical myelopathy (DCM) is a common progressive disease of the spinal cord which can cause tetraplegia. Despite its prevalence, few studies have investigated the pathophysiology of DCM. Macroautophagy is a cellular process which degrades intracellular contents and its disruption is thought to contribute to many neurodegenerative diseases. The present study tests the hypothesis that macroautophagy is impaired in DCM. To address this, we utilised a collection of post-mortem cervical spinal cord samples and investigated seven DCM cases and five human controls. Immunohistochemical staining was used to visualise proteins involved in autophagy. This demonstrated significantly reduced numbers of LC3 puncta in cases versus controls (p = 0.0424). Consistent with reduced autophagy, we identified large aggregates of p62 in four of seven cases and no controls. Tau was increased in two of five cases compared to controls. BCL-2 was significantly increased in cases versus controls (p = 0.0133) and may explain this reduction in autophagy. Increased BCL-2 (p = 0.0369) and p62 bodies (p = 0.055) were seen in more severe cases of DCM. This is the first evidence that autophagy is impaired in DCM; the impairment appears greater in more severe cases. Further research is necessary to investigate whether macroautophagy has potential as a therapeutic target in DCM.

## Introduction

Degenerative Cervical Myelopathy (DCM) is a common spinal cord disease caused by chronic spinal cord compression (SCC)^1^. The clinical phenotype is variable, with motor, sensory or autonomic symptoms in the limbs or torso possible^1^. Symptoms in mild DCM often remain stable over prolonged periods of time but can progress to moderate/severe forms of DCM with symptoms that can include tetraplegia and a devastating impact on quality of life^2–4^.

Disease modifying treatment is restricted to decompressive surgery in severe/moderate forms of DCM^5^. Surgery has been shown to halt disease progression and offer meaningful, albeit incomplete recovery^6^. This relates to the limited regenerative capacity of the spinal cord. The development of adjuvant neuroregenerative/neuroprotective therapies is thus a priority, but is hindered by a poor understanding of DCM pathogenesis. Two observations are critical. First, there is little correlation between the amount of spinal cord compression and disease severity^1^. Second, genetic associations indicate that spinal cord-intrinsic factors may be key to the progression of disease^7^.

Only around fifty articles have been published investigating the pathophysiology of DCM in preclinical models; they implicate a number of mechanisms including chronic ischaemia, apoptosis and macroautophagy^8,9^. Macroautophagy (henceforth autophagy) is an important mechanism of cell homeostasis that leads to the lysosomal degradation of cytoplasmic contents including tau, damaged organelles, and aggregate-prone proteins^10^.

Autophagy proceeds through an ordered sequence of events. Translocation of a multi-protein complex containing beclin-1 to nascent phagophore membranes is one of the events that enables autophagosome formation^10^. B cell lymphoma 2 (BCL-2) is a negative regulator of beclin-1 and hence inhibits autophagy^11^. Conjugation of microtubule-associated protein light chain 3 (LC3) to phosphatidylethanolamine on phagophore membranes converts LC3-I to LC3-II and is a key event in autophagosome formation^10^. Specific cytoplasmic contents are recruited into autophagosomes by cargo receptors such as p62 and degraded on fusion with lysosomes^10^. Impaired autophagy leads to accumulation of potentially toxic protein aggregates such as tau or damaged mitochondria; neurons are particularly sensitive to autophagy impairment^12^.

Animal models of chronic SCC identified improved neuronal survival following autophagy induction^13,14^. In patients with DCM, degeneration of neurons and glia has been associated with accumulation of tau aggregates^15^. However, autophagy has not yet been studied in humans with DCM in part because human spinal cord is rarely obtained at post-mortem.

Based on its important homeostatic role in neurons and pre-clinical studies we hypothesised that autophagy may be impaired in DCM, and that this impairment may be more pronounced in more severe cases of DCM. To address this hypothesis, we used a unique collection of human spinal cord samples for immunohistochemistry in order to investigate the expression of different proteins involved in autophagy in post-mortem human cases and controls of DCM^16^.

## Material and methods

### Patients

Cervical spinal cord samples (seven cases, five controls) were obtained with informed consent from the patients’ families prior to autopsy examination as previously reported^16^. Cases were diagnosed clinically and pathologically and managed conservatively without surgery. Tissue was stored according to institutional ethics guidelines in a facility licensed by the Human Tissue Authority.

### Staining

Cervical spinal cords were paraffin embedded and sectioned onto slides at a thickness of 6 μm. In cases, staining was undertaken in the sections of maximal compression as identified by gross morphological assessment. In controls a random cervical spinal cord section was stained. Due to limited tissue availability, a maximum of one section was stained with each antibody per individual. Deparaffinized sections were subject to antigen retrieval in 98% formic acid for 5 min followed by 4% aqueous hydrogen peroxide to block endogenous peroxidases. Sections were then rinsed with tap water and phosphate buffered saline (PBS) before being blocked with normal rabbit serum (Dako) in 20% PBS. Sections were then incubated with primary antibodies to phosphorylated tau (Thermo Scientific, 1/500). Other sections were subject to antigen retrieval in citrate at pH 6 and incubated with antibodies to BCL-2 (Dako, 1/100), LC3A/B (Cell Signalling Technology, 1/250), NeuN (Abcam, 1/1000) and p62 (BD Transduction Laboratories, 1/75). After rinsing for 5 min in PBS sections were incubated with secondary antibody (Rabbit Anti-Mouse 1/200, Dako) for 30 min. After rinsing for 5 min in PBS they were incubated in avidin–biotin complex (ABC, Vector) for 30 min before being developed with diaminobenzidine (DAB, Vector). Slides were then lightly counterstained with haematoxylin. Histological staining with luxol blue was also performed. Staining was undertaken by the Cambridge Brain Bank.

### Acquisition

Images were acquired using an Axioimager Z2. Brightfield images were obtained with the aperture at 0.21, LED intensity of 15–25% and an exposure time of 3-10 ms. The field was focussed at X20 or × 10 and the tiling program used to create images of the entire grey matter of both sides of the spinal cord, excluding the central canal and the majority of the grey commissure. Microscope settings were identical across samples for any given stain. Where available, the individual section stained with each antibody in each case and control was imaged. Images were exported as TIFF files with no compression.

### Analysis

The individual image for each antibody staining in every case and control was analysed. Colour deconvolution using the H DAB programme was used to isolate DAB staining. The grey matter was manually selected using the freehand area selection. The image was then thresholded to isolate actual DAB staining; the same threshold value was applied to all images stained with the same antibody. The threshold value was determined by manually comparing thresholded images to normal images for each stain. After thresholding, particle analysis was performed to determine the number, area and average size of staining. Except where stated, only discrete areas of staining above 0.25 μm^2^ were measured in an attempt to limit non-specific background staining or focussing errors distorting the results. Circularity was only used to limit analysis when investigating discrete areas of staining under 0.25 μm^2^. NeuN and p62 analysis involved the same process but larger areas of staining were also investigated—areas over 51.5 μm^2^ for p62 and areas over 23.3 μm^2^ and 101 μm^2^ for NeuN. LC3 puncta analysis differed only in the criteria used in particle analysis. Here, only discrete areas from 0.2–7.7 μm^2^ with a circularity over 0.99 were analysed. These criteria were used as they correlate with published sizes of autophagosomes and the expectation of their circularity in a two dimensional plane^17^. The evident gross deformity of cases compared to controls prevented blinding.

### Statistical analysis

In all analyses all usable data were included—some analyses did not include all twelve subjects due to damage to sections and the lack of tissue available. Distribution normality was assessed using the Shapiro–Wilk test and manual assessment of distribution. While the power of the Shapiro–Wilk test is limited by the low number of samples in our study, we considered non-parametric tests in those with values under 0.1. After assessment of data normality, two-tailed t-tests or Mann–Whitney tests were performed. Statistical analysis of correlations was performed using non-linear regression to create r^2^ and p values. Pearson correlation analysis was used to determine statistical significance of correlations.

### Ethics approval

Acquisition and analysis of autopsy specimens was approved by the institutional review board of Niigata University School of Medicine, Niigata, Japan. Informed consent was obtained from the patients’ families prior to autopsy examination. Existing tissue was transported from Japan for this study under Material Transport Agreement RG87826. Tissue was stored according to institutional ethics guidelines in a facility licensed by the Human Tissue Authority (Anne Mclaren Laboratory, Cambridge, UK). The research was conducted under the framework of the human tissue Act 2004.

### Consent for publication

All authors have consented for publication of the article.

## Results

### Study participants

The study included post-mortem samples from seven cases of DCM and five controls. Patients on average were 68.1 years old (range 61–80); controls had an average age of 68 (range 51–83). Clinical details are given in Tables 1 and 2 and causes of death in Supplementary Tables 1 and 2.

**Table 1:** Characteristics of cases of DCM.

Case	Sex	Age	Duration of illness (years)	No. of symptom groups	Severity	Other central nervous system (CNS) pathology
1	M	80	Unknown	1	Mild	Cerebral Infarct
2	M	70	1.3	2	Mild	
3	M	63	10	3	Mild	Dementia
4	M	59	8	4	Severe	Cerebral Infarct
5	M	72	2.8	4	Severe	
6	F	72	4	5	Severe	
7	M	61	2	6	Severe	Brain stem neuroma

The six symptom groups assessed were: upper limb weakness, upper limb sensory disturbance, trunk sensory disturbance, lower limb sensory disturbance, gait disturbance, exaggerated deep tendon reflex. Cases were classed as mild if they suffered from 3 or less symptom groups, and severe if they suffered 4 or more symptom groups.

**Table 2:** Characteristics of controls.

Control	Sex	Age	CNS pathology
1	F	73	Nil
2	F	83	Nil
3	F	68	Nil
4	M	65	Malignant lymphoma in cerebrum
5	M	51	Nil

Details of the five controls used in this study.

### Reduction of NeuN staining in DCM suggesting reduced neuronal size

**Figure 1:**
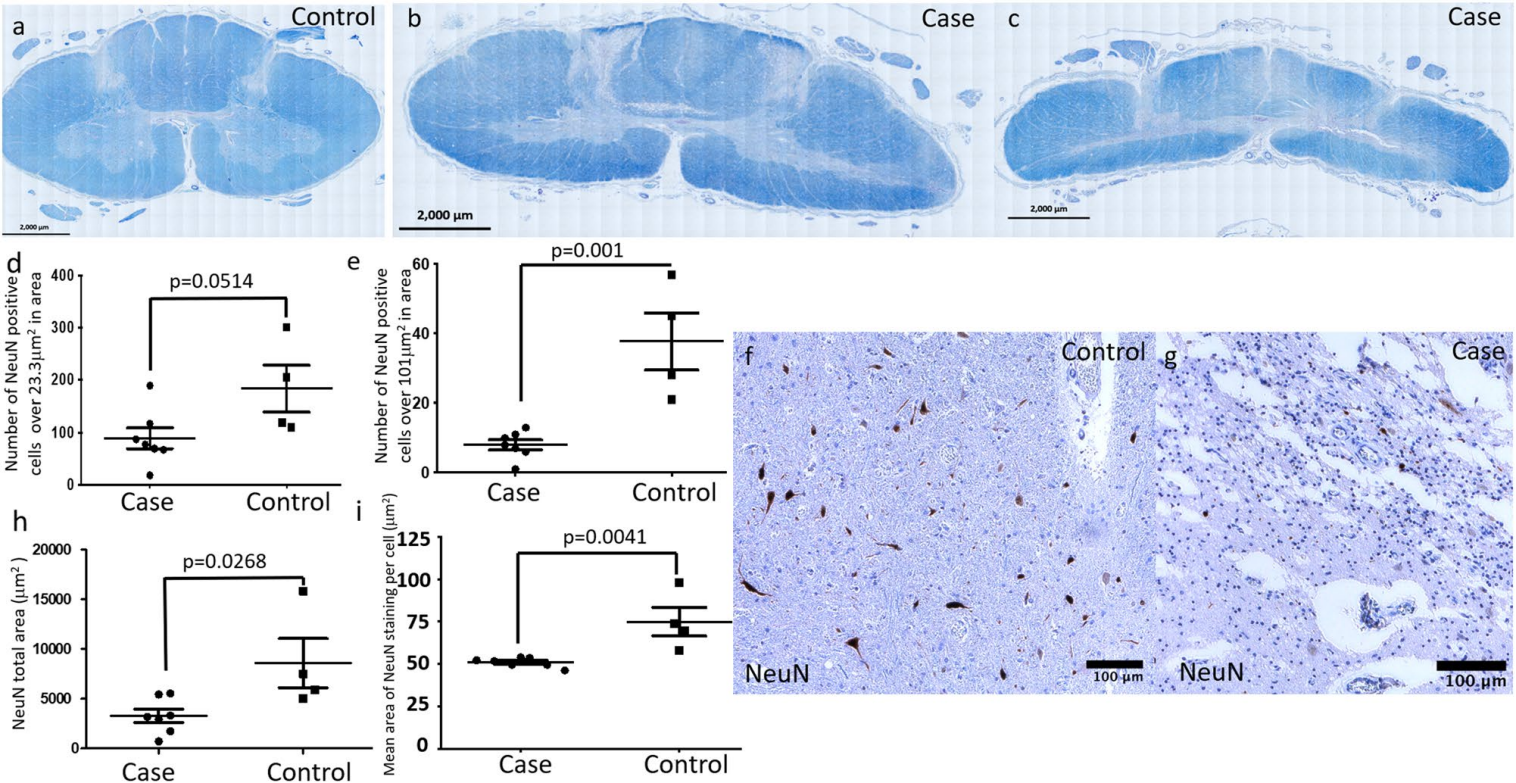
(**a**) Luxol blue staining of control spinal cord. (**b, c**) Luxol blue staining of spinal cord of two DCM patients demonstrating generalised compression and elongation with deformity particularly affecting the grey matter. (**d**) There was no difference in the number of NeuN positive discrete areas of staining over 23.3 μm^2^ in cases (n = 7) compared to controls (n = 4). (**e**) When assessing the number of NeuN positive discrete areas of staining over 101 μm^2^ there was a reduction in number in cases (n = 7) compared to controls (n = 4). (**f**) Control sample NeuN staining of grey matter at high magnification. (**g**) NeuN staining of grey matter at high magnification in one case. (**h**) There was a reduction in the total area of NeuN staining (including all discrete regions of staining over 0.25 μm^2^) in cases (n = 7) versus controls (n = 4). (**i**) There was a reduction in the mean size of NeuN positive regions when assessing areas of staining over 23.3 μm^2^ in area. Two tailed t-tests were performed for all graphs.

We first assessed the structure of the whole spinal cord sections. Luxol Blue staining revealed deformity and loss of tissue in the spinal cord white and grey matter in all cases (Fig. 1b, c) compared to controls (Fig. 1a). Previous animal models identified greater changes in autophagy in the grey matter leading to a focus on the grey matter in this study. To identify neurons, we performed NeuN staining in controls (Fig. 1f) and cases (Fig. 1g). NeuN is specifically located in neurons and has previously been used to assess neuronal size and shape^17^. The number of discrete areas of NeuN staining over 101 μm^2^ was reduced in DCM compared to control tissue (Fig. 1e), but the number of discrete areas of NeuN staining over 23.3 μm^2^ was comparable (Fig. 1d).Furthermore, the total area of NeuN staining was reduced in DCM (Fig. 1h), and the mean area of NeuN staining per cell (when analysing discrete regions over 23.3 μm^2^) was reduced (Fig. 1i). The % area of NeuN positive staining was unaltered between cases and controls (Sup Fig. 1a).

### LC3 puncta are reduced in DCM suggesting reduced autophagy

**Figure 2:**
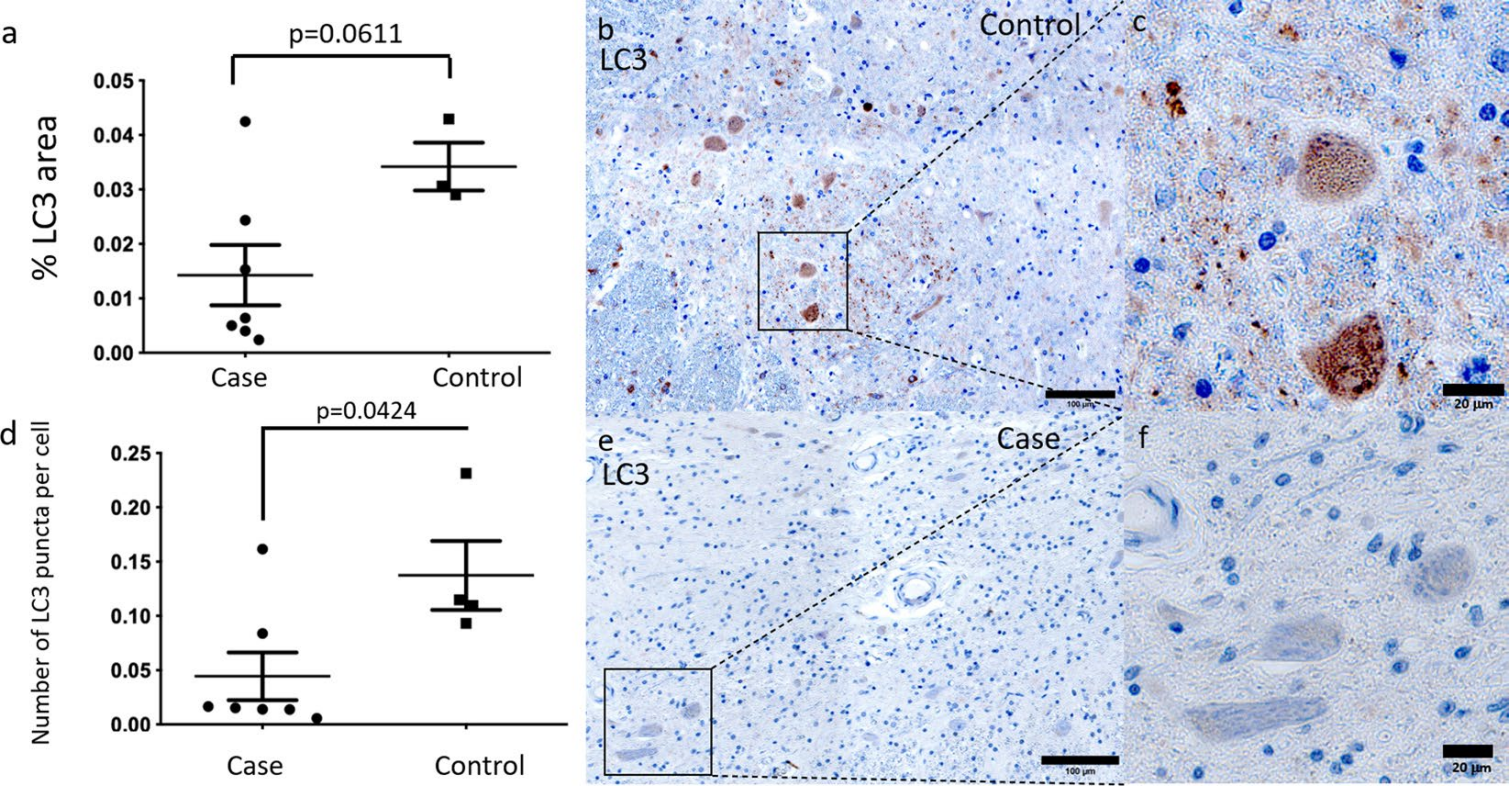
(**a**) Differences in the total % area of LC3 staining in cases (n = 7) and controls (n = 4) excluding regions below 0.25 μm^2^ did not reach statistical significance. (**b, c**) Control sample LC3 staining of grey matter at high magnification. (**d**) Number of LC3 puncta per cell (regions 0.2–7.7 μm^2^ in size with a circularity over 0.99 divided by the number of nuclei counted by isolating nuclear staining) in the grey matter was significantly lower in cases (n = 7) than controls (n = 4). (**e, f**) LC3 staining of grey matter in one case at high magnification. Two-tailed t-tests were performed in (**a**). Two-tailed Mann–Whitney tests were performed in (**d**).

A commonly used method for monitoring autophagy is to assess the presence of LC3^19^. We found that the total % area of LC3 staining was non-significantly lower in DCM (Fig. 2a, b, e). However, only LC3-II is directly involved in autophagy and is represented by LC3 punctate staining^20^. We found that the number of LC3 puncta per cell were significantly reduced in DCM cases compared to controls (Fig. 2c, d, f).

### The cargo receptor p62 and the substrate tau are increased in a subset of cases

**Figure 3:**
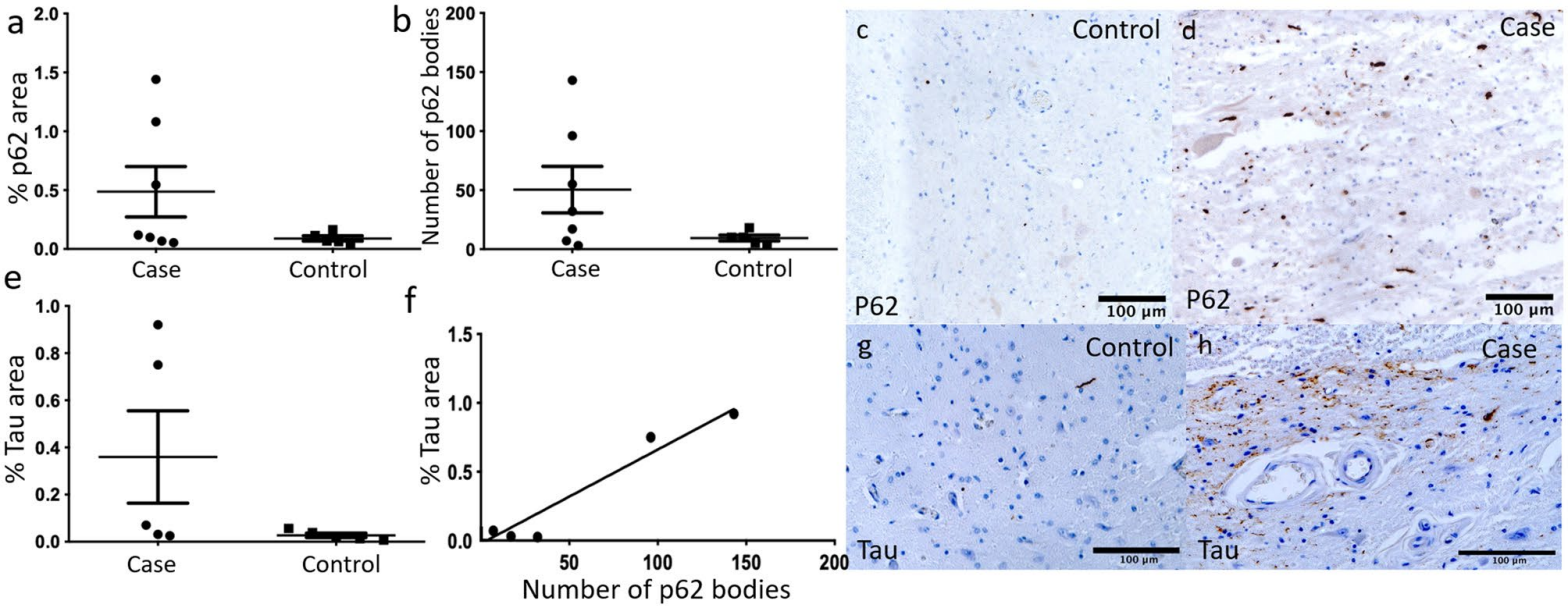
(**a**) Total area of p62 staining was increased in 3 cases (n = 7) compared to controls (n = 5) excluding regions below 0.25 μm^2^. (**b**) Number of p62 bodies with an area over 51.5 μm^2^ was elevated in 4 cases (n = 7) compared to controls (n = 5). (**c**) Control sample p62 staining of grey matter at high magnification. (**d**) p62 staining of grey matter in one case at high magnification. (**e**) Total area of tau staining was increased in 2 cases (n = 5) compared to controls (n = 5) excluding regions below 0.25 μm^2^ in cases (n = 5) and controls (n = 5). (**f**) XY graph of %tau area against number of p62 bodies; (**g**) control sample tau staining of grey matter at high magnification; there was no statistically significant correlation. (**h**) Tau staining of grey matter in one case at high magnification. Two-tailed Mann–Whitney tests were performed in (**a–c**). In (**f**), correlation was assessed using non-linear regression and statistical significance determined using Pearson correlation.

To further explore changes in autophagy, we stained for the autophagy receptor p62 and the autophagy substrate tau, both of which accumulate on autophagy inhibition. In the grey matter, the area of p62 was increased in 3/7 cases (Fig. 3a, c, d). In four of seven cases, we detected increased large p62 bodies (Fig. 3b). The area of tau staining was increased in two of five cases (Fig. 3e, g, h). The two cases with high Tau expression also had high p62 expression (Fig. 3).

### The autophagy inhibitor BCL-2 is increased in DCM and BCL-2 and p62 are correlated with increasing severity of DCM

**Figure 4:**
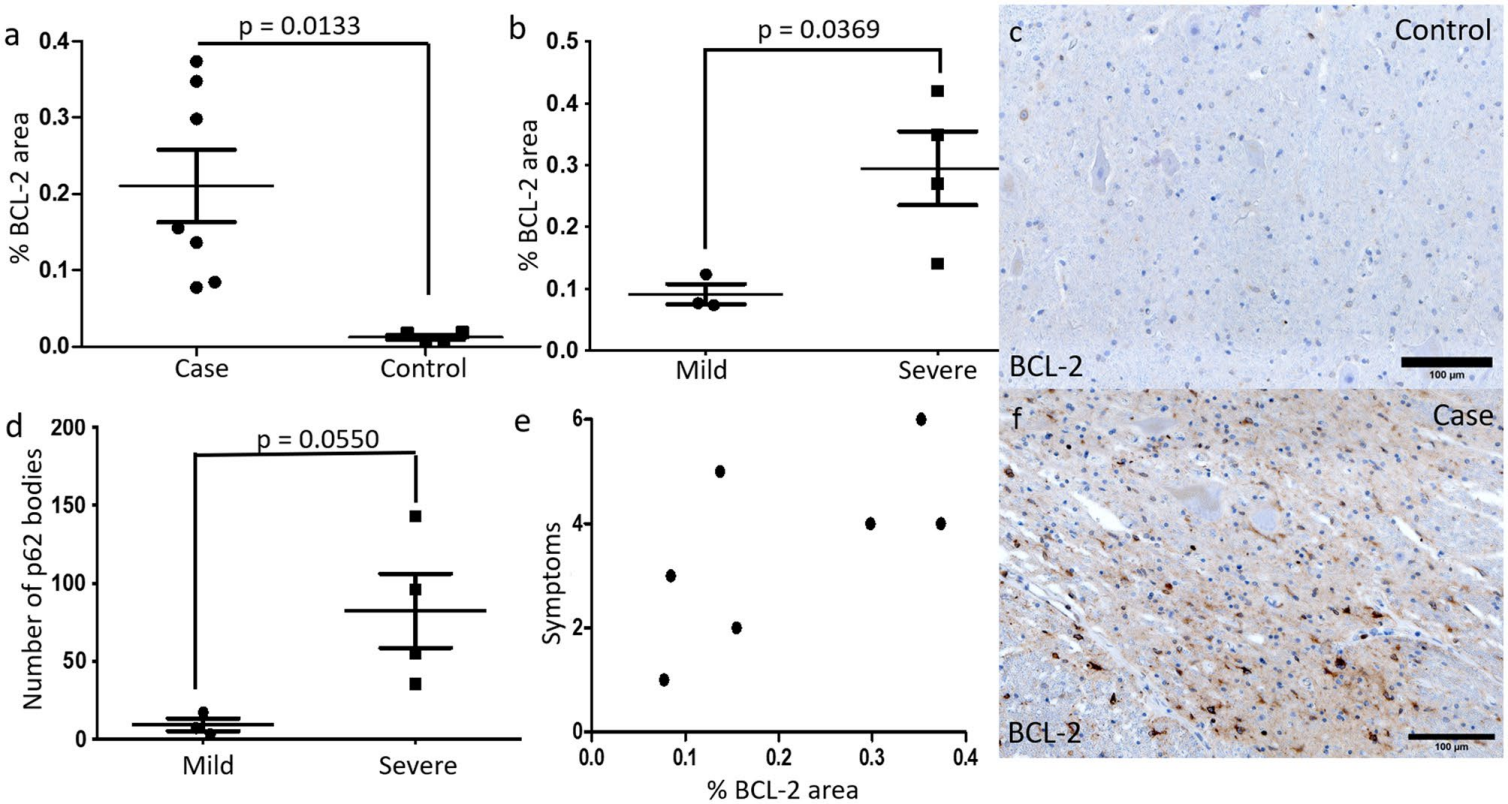
(**a**) Total area of BCL-2 staining was increased in cases (n = 7) compared to controls (n = 5) excluding regions below 0.25 μm^2^. (**b**) The total area of BCL-2 staining was significantly increased in severe cases (n = 4) compared to mild cases (n = 3). (**c**) Control sample BCL-2 staining of grey matter at high magnification. (**d**) The number of p62 bodies was increased in severe cases (n = 4) compared to mild cases (n = 3). (**e**) BCL-2 area correlated non-significantly with the symptomatic severity of cases. (**f**) BCL-2 staining of grey matter in one case at high magnification. Two-tailed t-tests were performed in (**a, c, d**). In (**e**), correlation was assessed using non-linear regression and statistical significance determined using Pearson correlation.

To investigate which factors might explain the reduction of autophagy seen in DCM cases, we assessed the expression of BCL-2 (Fig. 4a, c, f), an inhibitor of autophagy. We found a statistically significant increase in the area of BCL-2 staining in DCM as compared to controls (Fig. 4a). BCL-2 was positively but non-significantly correlated with the number of large p62 bodies (Sup Fig. 1d), but not the number of LC3 puncta per cell (Sup Fig. 1c). p62 also did not correlate with the number of LC3 puncta per cell (Sup Fig. 1b).

To identify any clinico-histological correlations, we compared the staining pattern identified in each patient with their clinical phenotype. We found that the number of large p62 bodies and the area of BCL-2 staining was higher in cases with severe disease compared to mild disease (Fig. 4b, d). Symptomatic severity non-significantly correlated with BCL-2 area expression (Fig. 4e). No other factors were significantly correlated with symptomatic severity (Sup Fig. 2).

## Discussion

We conducted the first investigation of autophagy in DCM through assessment of compressed cervical spinal cord sections in cases and normal cervical spinal cord sections in controls. This revealed a reduction in the number of LC3 puncta, suggesting a defect in macroautophagy. Consistent with reduced macroautophagy, we detected accumulation of large p62 bodies in four of seven cases and an increased area of Tau staining in two of five cases. We found increased BCL-2 staining which may explain the observed reduction in macroautophagy. Importantly, we found increased BCL-2 and p62 in more severe cases of DCM. Taken together, our data implicate defective macroautophagy at the level of spinal cord compression in the pathogenesis of DCM.

The reduction of LC3 puncta in cases suggest that autophagy is impaired in DCM. This finding contrasts the results of a mouse model of chronic spinal cord compression, which identified raised LC3-II^13^. The most likely explanation for this discrepancy relates to disease duration; the mice had motor paresis for eight weeks whereas the cases in this study had a diagnosis of DCM for up to eight years before death.

The observed impairment of autophagy may contribute to the pathogenesis of DCM by permitting accumulation of toxic aggregates or damaged mitochondria^21^. The present study identified accumulation of dangerous bodies such as p62 and tau in some patients with DCM further indicating defective autophagy. Importantly, our results implicate accumulation of large p62 bodies with symptomatic disease progression. Four cases were classed as severe and had an increased number of p62 bodies compared to mild cases. Furthermore, two different animal models of DCM have identified elevated p62 suggesting its involvement in the pathophysiology of DCM^13,14^.

The precise role of p62 in DCM remains unclear. In a *Drosophila* model of polyglutamine disease, depletion of p62 accelerated neurodegeneration^22^. Therefore, increased p62 could reflect a protective mechanism aimed at degrading toxic substances. However, the presence of p62 bodies could be directly pathogenic: overexpression of p62 induces impairment of mitochondrial function and neuronal loss^23^. Given its association with disease severity, the role of p62 in DCM requires further investigation.

We found significantly increased BCL-2 expression at the level of spinal cord compression in DCM, moreover, BCL-2 was significantly higher in severe cases compared to mild cases. BCL-2 was first identified as an inhibitor of apoptosis and upregulation of BCL-2 reduces neuronal cell death^24,25^. However, more recently BCL-2 has also been shown to inhibit autophagy possibly explaining the impaired autophagy identified in this study.

Unfortunately, animal models have shed little light on the role of BCL-2 in DCM. Two mouse models of DCM found a reduction and no change in BCL-2 respectively^26,27^. However, experimental cord compression for 5 min in rats induced BCL-2 expression for four days^28^. Further research is necessary to reconcile this conflicting data.

We also identified a reduction in the number of discrete NeuN positive regions over 101 μm^2^ suggesting a decrease in neuron size at the level of spinal cord compression. This is consistent with the observed molecular changes as cellular processes including autophagy regulate cell size^29^. The significance of this to neuronal function remains unclear.

Induction of autophagy is a possible therapeutic target in DCM. Lithium induces autophagy and its application in a mouse model of chronic spinal cord compression reduced p62 and increased LC3-II expression promoting neuronal survival^13^. The present study provides further weight to exploring this possibility. In addition, identification of features on magnetic resonance imaging or electrophysiological studies which correlate with histological indicators of impaired autophagy may highlight patients most likely to benefit from autophagy induction.

There are some limitations to this study. The small sample size and limited tissue availability and quality (some samples were destroyed in processing) meant that we were unable to include all twelve individuals in all experiments, limiting the statistical power of this study. Furthermore, the tissue was only amenable to histological and immunohistochemical staining. Nevertheless, these analytical techniques have been used to investigate autophagy^19,30^.

To conclude, we have provided the first evidence that macroautophagy is impaired in humans with DCM; this impairment appears greater the more severe the symptoms. Our results implicate defective macroautophagy in the symptomatic progression of DCM; inducing autophagy may therefore be a neuroprotective target in DCM.

## Supplementary Information

Supplementary Information.

## Data Availability

The datasets generated during and/or analysed during the current study are available from the corresponding author on reasonable request.
