# Supplementary material for: Evidence of impaired macroautophagy in human degenerative cervical myelopathy
Source: Sci Rep. 2022 Jul 13;12:11873. doi: 10.1038/s41598-022-15158-x (PMC9279443; doi:10.1038/s41598-022-15158-x)
Supplement: Supplementary file 1 — Supplementary Information. [file 41598_2022_15158_MOESM1_ESM.pdf]

Title: Evidence of impaired macroautophagy in human degenerative cervical myelopathy

Running title: Autophagy in Degenerative Cervical Myelopathy

Sam S. Smith[3], Adam M.H. Young[4], Benjamin M. Davies[1], Hitoshi Takahashi[4], Kieren S.J. Allinson[5], Mark R.N. Kotter. [1-2]

1) Academic Neurosurgery Unit, Department of Clinical Neurosurgery, University of Cambridge, Cambridge, UK
2) WT MRC Cambridge Stem Cell Institute, Anne McLaren Laboratory, University of Cambridge, UK
3) School of Clinical Medicine, University of Cambridge, Cambridge, UK
4) Department of Pathology, Brain Research Institute, Niigata University, Niigata, Japan
5) Cambridge University Hospitals NHS Foundation Trust and the Cambridge Brain Bank, Cambridge, UK

Supplementary Figures

Case	Sex	Age	Cause of death
1	M	80	Extensive cerebral infarction
2	M	70	Disseminated visceral giant cell arteritis
3	M	63	Colon cancer
4	M	59	Sudden unexpected death
5	M	72	Chronic renal failure
6	F	72	Peritonitis
7	M	61	Pneumonia

Supplementary Table 1. Causes of death for the 7 cases of DCM.

Control	Sex	Age	Cause of death
1	F	73	Thrombotic Thrombocytopenic purpura
2	F	83	Myasthenia Gravis
3	F	68	Facioscapulohumeral Muscular Atrophy, Aspiration Pneumonia
4	M	65	Malignant Lymphoma, Pneumonia
5	M	51	Acute abdomen

Supplementary Table 2. Causes of death for the 5 controls.

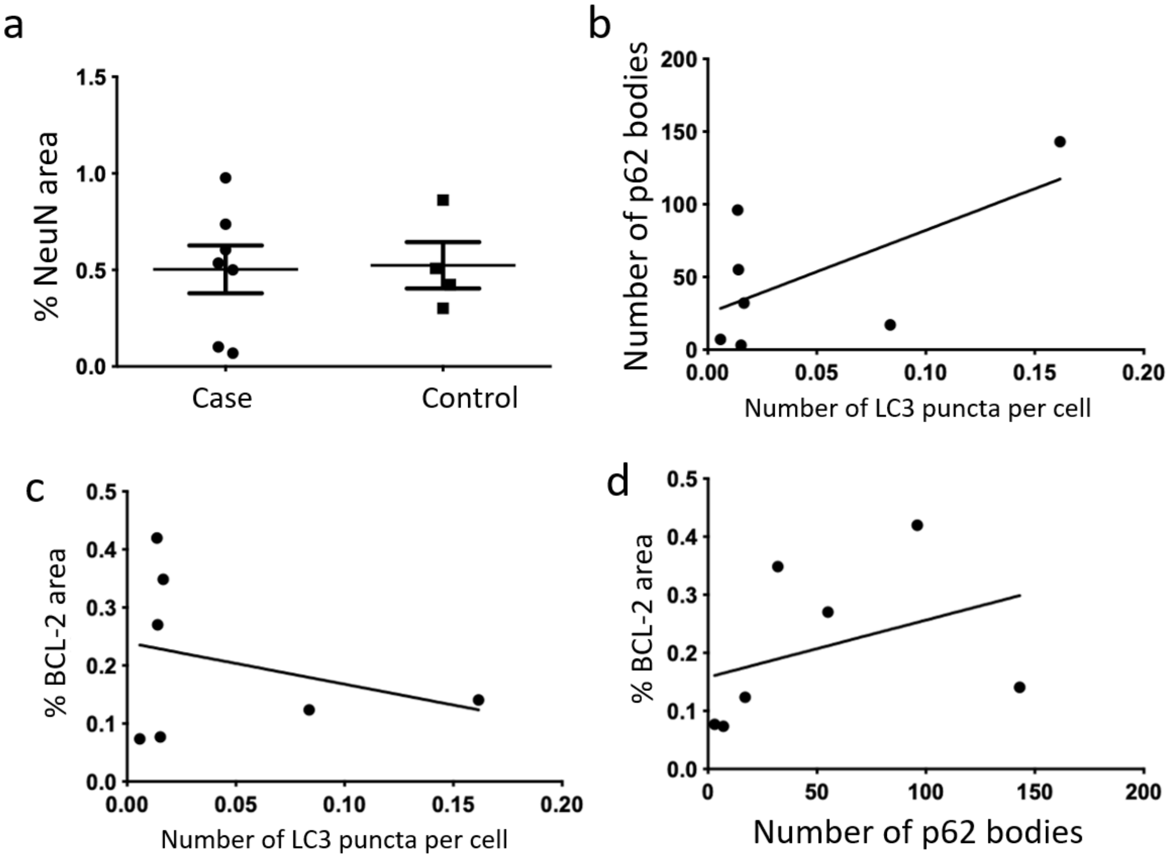

Supplementary Figure 1. A) % NeuN area of staining in cases compared to controls. b-d) XY Graphs comparing the relationship between different stains in cases. Correlation was assessed using non-linear regression and statistical significance determined using Pearson correlation. No correlations reached statistical significance.

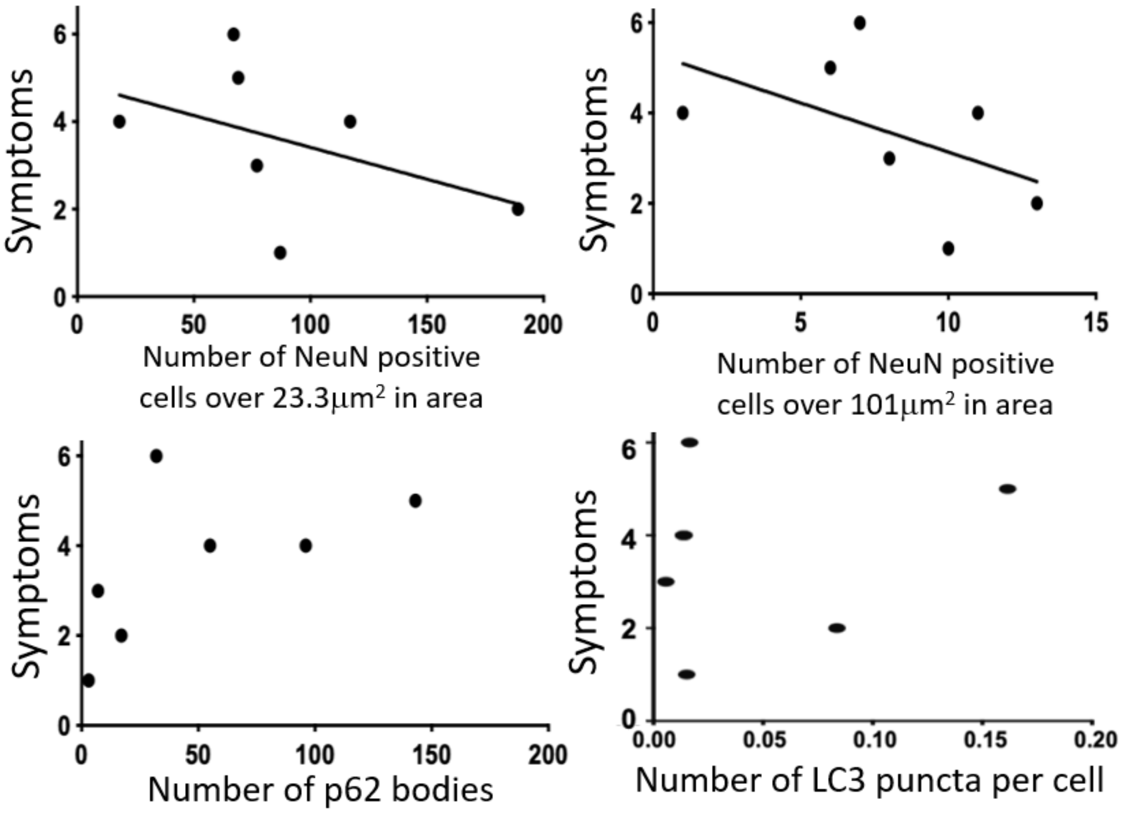

Supplementary Figure 2. A-d) XY graphs of cases (n=7) comparing the number of symptom groups against the results of analyses of staining for NeuN, p62 and LC3 earlier in this study. Correlation was assessed using non-linear regression and statistical significance determined using Pearson correlation. No correlations reached statistical significance.
